# Host Resistance and Hospital Infection

**Published:** 1969-04

**Authors:** W. A. Gillespie


### HOST RESISTANCE AND HOSPITAL INFECTION

BY

W. A. GILLESPIE

Hospital infections and methods of preventing them have been studied a great deal in recent years. Progress has been made but it has been one-sided. Much has been learned about the behaviour of the microbes that cause infection in hospitals and this epidemiological knowledge has helped to reduce cross-infection by organisms from outside the patient. Thus, for example, advances have been made in preventing airborne infection in operating theatres and in excluding extraneous bacteria from surgical wounds, from babies' skin, and from the urinary tract (Report, 1968). However, this progress in methods of controlling the organisms underlines our ignorance and lack of progress with the resistance of the patient and the factors that influence it.

Host resistance should not be thought of only in terms of defence against the virulent extraneous pathogens that assail the body from time to time. A state of latent infection is natural in everyone—an equilibrium between the body's defences and the many microbes that may cause overt clinical disease only when the resistance of the host is lowered. In hospital, many patients have lowered resistance and consequently are liable to suffer from infection. Some of the infections in these 'high-risk' patients are caused by extraneous organisms, and some are caused by the patient's own endogenous microbes, many of them commensals that behave as opportunistic pathogens. In some patients, resistance is lowered because of their age; in some it is lowered by the diseases that bring them into hospital; and in an increasing number it is lowered by the treatment that they are given.

There are many examples of increased susceptibility to infection at the extremes of life. Newborn babies are unduly liable to suffer from infection. The elderly tend to get pneumonia; and recent surveys have confirmed that old people are unduly susceptible to post-operative wound infection (Report, 1968).

Of the diseases that increase susceptibility to infection, diabetes is perhaps the best known; but this statement has to be qualified because some surveys of wound infection and urinary infection have failed to reveal a higher incidence in diabetics than in other people. However, there is no doubt that infections tend to be more serious in severe diabetes. Some infections in diabetics are caused by pathogens such as *Staphylococcus aureus,* but others are caused by organisms that rarely harm normal people. This was well illustrated in a series of infections in diabetics studied in Bristol (Wills & Reece, 1960). These were fatal cases of cellulitis of the lower limb, accompanied by much gas production, resembling gas gangrene. However, the bacteria were not clostridia but *Bacteroides species, Streptococcus fæcalis* and coliforms, organisms that would not behave like this in normal tissue.

Other diseases that cause increased susceptibility are the malignant reticuloses. Nowadays patients are kept alive longer, and their treatment often causes further damage to their defence mechanisms. In consequence, they sometimes provide striking illustrations of infections provoked by lowered host resistance. Some of the infecting organisms are high-grade pathogens like *Staph. aureus;* but others, the majority, are opportunists, either from the environment (*Aspergillus,* for example), or commensals from the patient himself. These commensals include such bacteria as *Achromobacter* and *Serratia, Candida* species, and cytomegalovirus. A good example was described recently by Symmers (1965). A patient with advanced Hodgkins disease had been treated with X-rays and a succession of cytotoxic drugs. Post mortem examination revealed infection by seven different organisms including staphylococci, *Candida, Aspergillus,* cytomegalovirus, and *Pneumocystis.* This showed what may happen when host resistance is badly deranged by a combination of a disease and its treatment—the wide range of microbial invaders, bacteria, fungi, viruses and protozoa; some from the environment but the majority probably endogenous; some of them recognised pathogens but others opportunists that would be incapable of infecting a normal host. In these circumstances the distinction between pathogen and non-pathogen breaks down. Some of the opportunists are so inconspicuous in the ordinary way that their very existence was only discovered in cases like this. For example, the protozoon *Pneumocystis* was recognised by finding it in pneumonia in premature and malnourished infants and in patients with hypogammaglobulinæmia or leukæmia, or during prolonged steroid therapy.

Iatrogenic causes of diminished host resistance are becoming increasingly prominent. Many of them are important, or even essential measures in the management of cancer, of auto-immune disease, and in organ transplantation. The iatrogenic causes of reduced resistance to infection are of two kinds: (i) local causes that lower the resistance of one tissue or area, and (ii) general causes, that lower the resistance of the whole body.

Local causes are well recognised. At the experimental level they are exemplified by the work of Elek and Conen (1957) who injected coagulase-positive staphylococci into the skin of healthy volunteers. In order to produce a visible lesion it was necessary to inject millions of cocci; but when the injection was given into skin near a foreign body (a suture), far fewer cocci caused sepsis. A similar effect of local tissue damage and foreign bodies is well known in clinical surgery. The liability of a wound to sepsis is increased with large wounds, with tissue damage and hæmatomata, and with drainage tubes (Report, 1968). The influence of a foreign body is most marked when it is left permanently in an organ—for example, the valve prostheses used in cardiac surgery. In a recent Swedish report, Lindbom and Laurell (1967) reported a 50 per cent incidence of wound and other sepsis after prosthetic replacement of diseased heart valves, compared with no infections at all in their series of mitral valvotomy operations. It is interesting that most writers report *Staphylococcus albus* as the commonest cause of post-operative endocarditis. This organism is a ubiquitous and common commensal, normally harmless. It is present in the air of occupied rooms, and on the skin; and so, when the distinction between non-pathogen and pathogen breaks down, it is not surprising that it often causes infection.

Good examples of diminished resistance to infection from local causes are found in the urinary tract. Any operation on urethra or bladder, or the use of a catheter, is likely to be followed by infection. Indwelling catheters are foreign bodies and also are potential channels of infection. When they are used the infection rate approaches 100 per cent unless great care is taken to maintain sterility. Since most of the risk is from organisms entering through the urethra or catheter, it is usually possible to prevent infection by using a combination of closed aseptic drainage and antiseptics, without recourse to antibiotics. By these means, infection rates after operations and other procedures involving the use of indwelling catheters were reduced from about 90 per cent to less than 20 per cent (Mitchell & Gillespie, 1964; Gillespie *et al.* 1967).

Many other local causes of diminished host resistance could be cited. Since extraneous organisms are often concerned, it has often been possible to prevent infection by excluding the organisms or to render the tissue hostile to them by means of antiseptics. The value of physical methods in conjunction with local antiseptics for excluding organisms and hence preventing infection in burns has been amply demonstrated (Cason *et al.* 1966). Work in Bristol and other centres leaves no doubt about the value of application of hexachlorophane to newborn babies' skin, in protecting them and their mothers from staphylococcal infection (Baber *et al.* 1967).

### GENERAL IATROGENIC CAUSES OF DIMINISHED RESISTANCE

More and more of these causes are operating in hospitals. They include the use of broad-spectrum antibiotics which eliminate the protective barrier of the normal body flora and so increase the risk of super-infection by organisms such as drug resistant staphylococci and *Pseudomonas pyocyanea*. Other general causes are cortico-steroid hormones, irradiation, cytotoxic and immuno-suppressive drugs, and anti-lymphocytic serum. As with the local causes, these general causes may increase the risk of infection by organisms from outside the body. But they are also liable to light up latent infections inside the body or to permit the patient's own commensals to invade his tissues. There are many experimental examples of these tendencies. For instance, cortisone treatment of rats leads to fatal endogenous infection by bacteria that normal rats harbour without harm (LeMaistre and Tompsett, 1952). The diminution of resistance caused by immuno-suppressive therapy extends to all kinds of organisms, not just bacteria. Taliaferro and Taliaferro (1948) showed years ago that resistance to malaria in chickens was reduced by irradiation and also by nitrogen mustard. Other experiments have demonstrated lowering of defences against viruses. Monkeys, normally resistant to smallpox virus, were infected fatally after treatment with 6-mercaptopurine, probably because of blocking of the primary antibody response (Janssen *et al.* 1962). Methotrexate was shown to inhibit antibody production in dogs infected with distemper virus. Attenuated distemper virus caused fatal infection in treated dogs, whereas normal animals suffered only a mild, short-lived infection (Thomas *et al.* 1963). Hence irradiation, and the chemotherapy that is used clinically to prevent graft rejection, may reduce resistance sufficiently to allow even benign organisms to cause dangerous infection. Woodruff (1966) reported that artificial immunisation of dogs to distemper may be interfered with by antilymphocytic serum. There is not yet much evidence about the extent to which ALS interferes with cellular immunity to bacteria, but Gaugas and Rees (1968) have recently demonstrated an enhanced susceptibility of mice to mycobacteria.

### MODE OF ACTION OF IMMUNO-SUPPRESSIVE DRUGS

Several different kinds of agent have this property. Some are alkylating agents (e.g. cyclophosphamide); some are pyrimidine or purine analogues, like 6-mercaptopurine or " Imuran "; some, e.g. methotrexate, act by blocking the transformation of folic acid and hence interfere with the biosynthesis of purines. All of them act, in one way or another, on proteins or the mechanisms of protein synthesis. Depending on how and when the drugs are administered in relation to antigenic stimulation, it seems that they can damage all the mechanisms, humoral and cellular, that make up acquired immunity (Berenbaum, 1964). They can interfere with the induction of immunity, the production of different classes of immuno-globulins, and with delayed hypersensitivity. Not all the mechanisms will be equally affected by a particular drug used in a particular way.

Irradiation also interferes with humoral and cellular immunity. It has been known for many years that the susceptibility to acute bacterial infection that follows radiation injury is associated with depressed leucocyte production by the marrow. Recent work (Smith *et al.* 1963) indicates that this is the important effect; the leucocytes that are produced function normally, but there are too few of them.

### METHODS OF PROTECTING HIGH-RISK PATIENTS

Everything possible should be done to exclude extraneous organisms and prevent cross-infection. This is more important than with ordinary patients. Preventive methods will include isolation from sources of infection; the rational use of disinfectants (see ' Notes on Disinfection ', 1968, available from the Office of the S.W. Regional Hospital Board, Tyndall's Park Road, Bristol); and, when appropriate, controlled ventilation or laminar air flow. But even if cross-infection is prevented, there remains the problem of endogenous infection by opportunists from the patient's alimentary canal, respiratory tract or other tissues. The principal safeguard must be to learn more about the mode of action of immuno-suppressive drugs and antilymphocytic serum in order to obtain the optimum effect on the patient's disease or on his homograft rejection, with the minimum disturbance of anti-microbial immunity.

*Prophylactic chemotherapy* has been widely used in surgical patients in the hope of preventing infection. Its place in the protection of high-risk patients clearly deserves consideration. This is not a simple matter. Prophylactic antibiotics have given disappointing results in surgery and urology, when given routinely as a kind of protective umbrella, with the intention of preventing infection by all kinds of bacteria. Several reports suggest that routine antibiotic prophylaxis does more harm than good (Report, 1968). This is not surprising, since no one drug can protect a patient against all the different bacteria that may infect him. The effect of using an antibiotic may be to substitute a resistant strain for a sensitive one. And whatever the drug, its widespread use will encourage resistant strains to multiply.

The situation is different, and more hopeful, if we aim at protecting a patient, not against all bacteria, but against one dangerous organism whose sensitivity we know. Thus, it is right to use penicillin to protect patients with rheumatic or congenital heart disease against endocarditis by *Streptococcus*

*viridans* during dental operations; this organism is usually penicillin-sensitive. The same antibiotic should be used to prevent clostridial infection after major operations on the lower limb (Parker, 1967). It is right to use an antibiotic to prevent complications of urological operations in patients whose urine is already infected; the correct drug should be chosen from the results of a recent bacteriological examination of the urine.

We must conclude that the value of prophylactic antibiotics is limited. When they are used, they should be used judiciously, in chosen patients, and generally for a short period, to help the patient to survive a dangerous episode.

### REFERENCES

Baber, K. G., Corner, B., Duncan, E. H L., Eades, S. M., Gillespie, W. A., Walker, S. C. B. (1967). J. Hyg., Camb., **65,** 381.

Berenbaum, M. C. (1964). Brit. med. Bull., **20,** 159.

Cason, J. S., Jackson, D. M., Lowbury, E. J. L., Ricketts, C. R. (1966). Brit. med. J., ii, 1288.

Elek, S. D., Conen, P. E. (1957). Brit. J. exp. Path. **38,** 573.

Gaugas, J. M., Rees, R. J. W. (1968). Nature, **219,** 408.

Gillespie, W. A., Lennon, G. G., Linton, K. B., Phippen, G. A. (1967). Brit. med. J. **3,** 90.

Janssen, R. J., Marshall, R. G., Gerone, P. J., Cheville, N. F. (1962). J. inf. Dis. **111,** 155.

LeMaistre, C. A., Tompsett, R. (1952). J. exp. Med. **95,** 393.

Lindbom, G., Laurell, G. (1967). Acta Path. Microbiol. Scand. **69,** 237.

Mitchell, J. P., Gillespie, W. A. (1964). J. Clin. Path. **17,** 492.

Notes on Disinfection and Sterilization Procedures (1968). Printed by S.W. Regional Hospital Board.

Parker, M. T. (1967). Brit. med. J. ii, 698.

Report to the Medical Research Council (1968) Lancet i, 705, 763, 831.

Smith, M. R., Fleming, D. O., Wood, H. B. (1963). J. Immunol., **90,** 914.

Symmers, W. St.C. (1965). Proc. roy. Soc. Med. **58,** 341.

Taliaferro, W. H., Taliaferro, L. G. (1948). J. inf. Dis., **82,** 5.

Thomas, E. D., Baker, J. A., Ferrebee, J. W. (1963). J. Immunol. **90,** 324.

Wills, M. R., Reece, M. W. (1960). Brit. med. J. ii, 566.

Woodruff, M. F. A. (1966). Lancet i, 980.

